# Running for Trains

**Published:** 1885-04

**Authors:** 


### Running for Trains.

We have just missed a train, and we missed it because we would not *run*.

This experience suggests to us to offer a few words of advice, through our readers, to the busy men of America who are in a chronic state of "*running for trains.*"

Even to one whose heart is sound, running, when not accustomed to such hurried movements, is certainly not beneficial to the delicate cords and valves of the heart; and should this organ be diseased, it must prove very injurious.

We all know that violent and tumultuous action is to be avoided when the heart is weak, and we also know that running is not the way to avoid it.

In our own experience we know several instances where men who had previously supposed themselves to be *sound* have run for trains, and getting aboard have fallen exhausted into seats from which they have been removed as corpses; and knowing such cases, we *never* run for trains.

Better miss a train than run the risk of running into the jaws of death; for this strain on the heart cannot prove beneficial to one that is sound, while it most positively will prove more or less disastrous to one that is weak. In this world of unsuspected physical pitfalls, it behooves us "*to make haste slowly.*" (Editorial.) —*Med. and Surg. Rep.*

